# Social media influenced self-application of cyanoacrylate for double chin reduction in an adolescent girl: an unusual case of post-inflammatory hyperpigmentation

**DOI:** 10.1097/MS9.0000000000001806

**Published:** 2024-02-08

**Authors:** Saurav Agrawal, Ved Prakash Pant, Anup Pandey, Sabin Acharya, Seema Sitaula, Aadesh Rayamajhi, Deepak Raj Pant

**Affiliations:** a Maharajgunj Medical Campus, Tribhuvan University, Institute of Medicine; b Department of Dermatology and Venereology, Tribhuvan University Teaching Hospital, Kathmandu; c B.P. Koirala Institute of Health Sciences, Dharan, Nepal

**Keywords:** adolescent, case report, cyanoacrylate, pigmentation, social media

## Abstract

**Introduction:**

Cyanoacrylate, used as a topical adhesive for wound closure in clinical settings, can result in poor cosmetic outcome on application to skin. Lack of formal medical or dermatological training among social media influencers poses risks of improper diagnosis, incorrect treatments, ineffective home remedies, and potential self-injury or long-term skin effects, especially among adolescents.

**Case presentation:**

The authors present a case of a young girl with a persistent post-inflammatory hyperpigmentation after using cyanoacrylate on her chin as a home remedy to reduce her double chin problem after learning from a video on social media. Biopsy findings were consistent with post-inflammatory hyperpigmentation in dermis.

**Clinical discussion:**

Application of cyanoacrylate over skin can result in allergic reactions, burn injuries, infections, itching, skin blistering, and aesthetic issues. Persistent post-inflammatory hyperpigmentation can be a poor cosmetic outcome on application of cyanoacrylate over skin.

**Conclusion:**

Inadequate social media safety regulations require healthcare professionals to be aware of social trends among adolescents and to encourage them for open conversations and professional help-seeking during times of distress in this digital era.

## Introduction

Highlights

- Application of cyanoacrylate can lead to persistent post-inflammatory hyperpigmentation on the skin.
- Inadequate social media safety requires healthcare professionals to stay informed about social trends among adolescents.
- Encouraging adolescents in open and non-judgmental conversations and to seek professional help is essential in this digital age.

Common pigmented dermatoses include post-inflammatory hyperpigmentation, ashy dermatosis, and pigmented purpuric dermatosis. Skin pigmentation can also result from metabolic conditions, exposure to metals, minerals, and drugs^1^. Cyanoacrylate, used as a topical adhesive for wound closure, can lead to various adverse effects, including allergic reactions, burn injuries, infections, itching, skin blistering, and aesthetic issues^2–4^. Social media has assumed a central role in influencing our choices, particularly when it comes to aspects of our lifestyle. Social networking platforms such as Facebook, Twitter, Instagram, and TikTok hold significant sway over decisions related to skincare, cosmetics, aesthetic procedures, and dermatological treatments. Around two-thirds of the influencers on these platforms lack formal medical or dermatological training, raising concerns about the potential for improper diagnosis, incorrect treatment suggestions, promotion of ineffective home remedies, and misguided management, which may inadvertently result in self-injury or long-term skin effects, particularly among adolescents^5,6^.

Inadequate social media safety requires healthcare professionals to stay informed about social trends among adolescents. Encouraging adolescents in open and non-judgmental conversations and to seek professional help is essential in this digital age. To the best of our knowledge, social media influenced self-application of cyanoacrylate leading to skin hyperpigmentation has not been reported in the literature yet. We present an unusual case of post-inflammatory hyperpigmentation in a 17-year-old girl who applied cyanoacrylate to her chin and lips after learning about it on social media as a solution for her double chin problem. This case has been reported in line with the SCARE 2023 criteria^7^.

## Case presentation

A 17-year-old young girl presented at the Dermatology outpatient department with a complaint of multiple black pigmented lesions on her chin and lips persisting for the past 5 months. The lesions developed after she applied cyanoacrylate as a home remedy to reduce her double chin problem. She had been concerned about her double chin and came across a video on social media suggesting the use of cyanoacrylate for double chin reduction. Fascinated by the possibilities presented by this unconventional approach, she chose to experiment with it firsthand. It was left on chin for 3 days and when she eventually removed it, she first noticed a red lesion on her chin that extended to involve both lips. Involved area was swollen, tender and pruritic. After 2 weeks of application, it changed to blackish discoloration that persisted for the past 5 months and caused her significant distress. She then applied herbal medicine but the lesion did not improve. She has no significant medical history, prior skin conditions or pigmentation abnormalities. Being an active social media user, she frequently relies on such platforms for beauty tips and advice. There is no known family history of skin disorders or pigmentation abnormalities.

**Figure 1:**
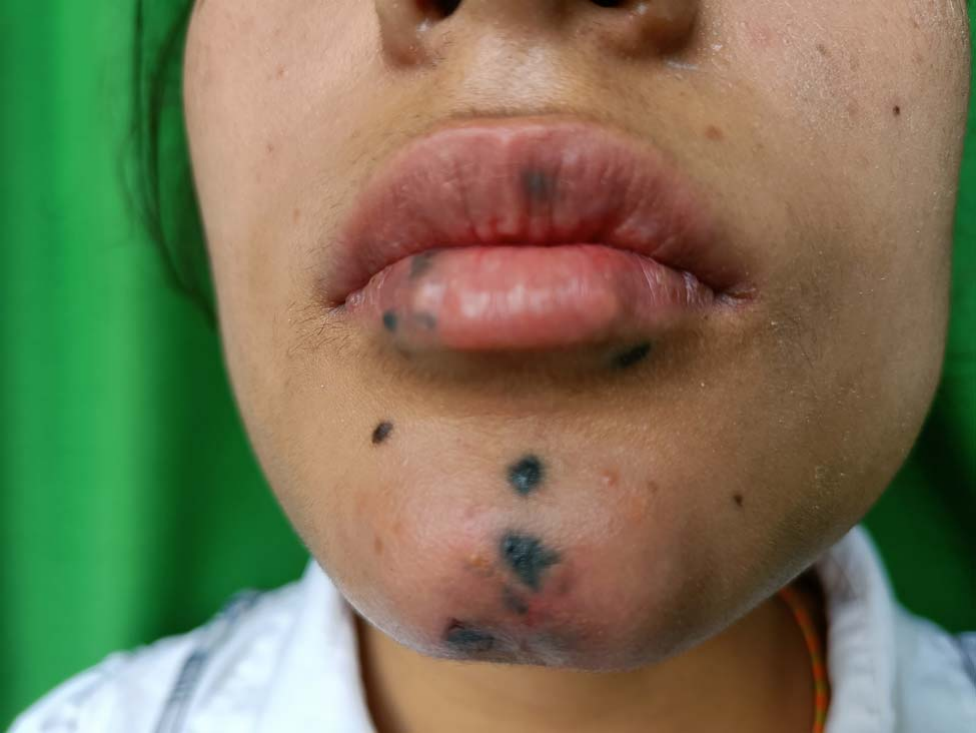
Multiple, black hyperpigmented macules of variable size, limited to area of chin and lips.

**Figure 2:**
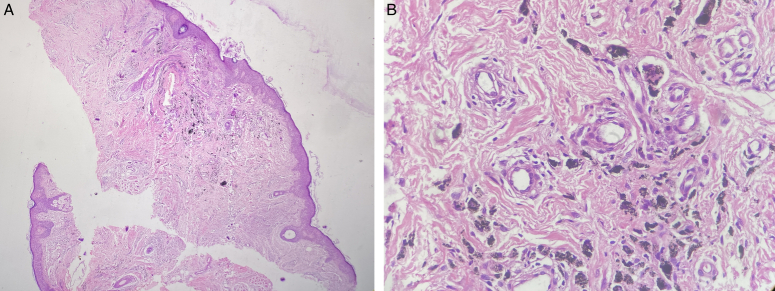
Histopathology showing multiple clumps of variable sized brown pigments in dermis with aggregates of macrophages engulfing the pigment. A: At 40 × magnification; B: At 400 × magnification.

Physical examination revealed multiple, black hyperpigmented macules of variable size, limited to area of chin and lips. The skin surrounding the lesions showed signs of mild irritation without significant inflammation or scarring (Fig. 1). Based on the patient’s clinical history and physical examination findings, the development of multiple pigmented lesions on the chin and lips are likely post-inflammatory outcomes after chemical irritation with cyanoacrylate. However, to confirm the diagnosis, biopsy of one of the lesions was performed that revealed multiple clumps of variable sized brown pigments in dermis with aggregates of macrophages engulfing the pigment (Fig. 2). These findings were consistent with post-inflammatory hyperpigmentation.

She was advised to discontinue use of cyanoacrylate or any other potentially harmful substances. She was prescribed topical depigmenting agent (hydroquinone 4%) and was educated about the risks and dangers associated with unconventional methods found on social media platforms for beauty purposes and to seek professional guidance for any health problems. Psychosocial support and counselling were provided to address her distress and concerns regarding her appearance. On follow-up after 6 months, there is gradual improvement of lesion with minimal residual pigmentation that is subsiding with its natural course.

## Discussion

Childhood and adolescence are marked by immaturity in foreseeing consequences, impulsivity, a focus on seeking social approval, which can result in poor decision-making. The rise of prevalent social media trends has escalated the risk of increased exposure to self-harm behaviours in this susceptible age group, driven by negative messages, emulation of self-injurious actions, and adopting self-harming practices from shared videos. A systematic review by memon and colleagues provides evidence that increased time spent on online social networking is associated with higher exposure to and involvement in self-harming behaviours. It also correlates with elevated psychological distress and suicidal thoughts in depressed adolescents^8^. Despite the growing reliance on social media for various purposes, it’s important to note that a substantial amount of unverified users without proper medical credentials post false and questionable information on these platforms^9^ that can lead to accidental self-injury or permanent skin changes, but they are typically not intended to cause harm. Instead, they offer the benefit of gaining social acceptance or becoming famous within one’s peer circle by sharing content on social media. Participating in these social media challenges can lead to burns, long-lasting skin discoloration, scarring, and infections. In the most extreme instances, some individuals have lost their lives due to these injuries^10^. In our case, we had an adolescent girl who was troubled by her double chin appearance. Rather than seeking professional advice, she opted to search for a solution on social media, where she came across a video posted by a non-medical individual suggesting the use of cyanoacrylate for double chin reduction in pursuit of social acceptance. However, this approach led to persistent post-inflammatory hyperpigmentation on her skin.

Cyanoacrylate is employed in clinical settings commonly for wound closure in cases with low tension surgical incisions, easily approximated traumatic skin lacerations, skin tears, and the closure of skin flaps and fragile skin. This choice of adhesive is favored due to its rapid and painless application, eliminating the need for local anaesthetic^11^. While these are usually well-tolerated, it can trigger a localized allergic reaction in 0.5–14% of patients^2^. There have also been documented cases of burn injuries to the skin in paediatric individuals resulting from accidental spills of cyanoacrylate-based adhesive, which necessitated skin grafting^3,4^. Other possible adverse effects of its use include infection, itching, skin blistering and less than optimal aesthetic outcomes such as post-inflammatory hyperpigmentation in our case.

Common pigmented dermatoses include post-inflammatory hyperpigmentation, which develops in response to inflammatory conditions; ashy dermatosis, a subtype of *lichen planus actinicus*; pigmented purpuric dermatosis, marked by the leakage of red blood cells and the accumulation of hemosiderin in the dermis. Furthermore, skin pigmentation can arise due to metabolic factors, including ochronosis, as well as exposure to metals like silver, mercury, lead, arsenic, zinc, bismuth, and iron. Additionally, minerals and certain medications can deposit pigments within the skin^1^. Post-inflammatory hyperpigmentation can result from various factors such as infections like dermatophytoses or viral exanthems, allergic reactions such as insect bites or contact dermatitis, papulosquamous diseases like psoriasis or lichen planus, medication-induced hypersensitivity reactions, and cutaneous injury from irritants, burns, or cosmetic procedures. Excessive melanin production or uneven pigment dispersion after cutaneous inflammation contributes to it. In the epidermis, increased melanin production and transfer to nearby keratinocytes occur, stimulated by factors like prostanoids, cytokines, chemokines, and inflammatory mediators, including reactive oxygen species released during inflammation. Dermal post-inflammatory hyperpigmentation, involving damage to basal keratinocytes, leads to the release of melanin, which is then phagocytosed by macrophages in the upper dermis, resulting in a blue-grey discoloration at the injury site^12^. In our particular case, the application of cyanoacrylate on the chin area induced an inflammatory reaction in the dermis that eventually resolved, leaving post-inflammatory hyperpigmentation, with supportive biopsy results.

Social media platforms have yet to widely establish effective regulations and policies to guarantee online safety and to curb the dissemination of potentially harmful content, including explicit descriptions of suicide or self-harm incidents. It is essential for health professionals to stay informed about the social trends prevalent in this age group and should maintain a vigilant awareness of any skin-related signs that might signal potentially risky behaviour on social media. Equally important is the need for parents, caregivers, and educators to closely supervise the online activities of young individuals, provide open and non-judgmental communication, educate them about online risks, and seek professional help in cases of severe distress. Additional research is needed to explore the safety and efficacy of non-professional cosmetic procedures promoted on social media and the psychological impact of facing unsatisfactory cosmetic outcomes, such as post-inflammatory hyperpigmentation.

## Conclusion

Application of cyanoacrylate over the skin can lead to persistent post-inflammatory hyperpigmentation with poor cosmetic results. Effective regulations on social media safety are lacking, making it crucial for healthcare professionals to stay informed about social trends among adolescents. Encouraging adolescents to participate in open and non-judgmental conversations, providing them with guidance on online risks, and encouraging them to seek professional help when experiencing significant distress is essential in this digital age.

## Ethical approval

Ethical approval is not required for a case report from the ethics committee at our institution.

## Consent

Written informed consent was obtained from the patients and parents for publication and any accompanying images. A copy of the written consent is available for review by the Editor-in-Chief of this journal on request.

## Source of funding

This research did not receive any specific grant from funding agencies in the public, commercial or not-for-profit sectors.

## Author contribution

S.A.: conceptualization, writing-original draft preparation, literature review, manuscript editing and review, and final manuscript approval. V.P.P.: conceptualization, literature review, manuscript editing and review, and final manuscript approval. A.P.: manuscript editing and review, and final manuscript approval. S.A.: manuscript editing and review, and final manuscript approval. S.S.: manuscript editing and review, and final manuscript approval. A.R.: manuscript editing and review, and final manuscript approval. D.R.P.: manuscript editing and review, and final manuscript approval.

## Conflicts of interest disclosure

Not Applicable.

## Research registration unique identifying number (UIN)

1. Name of the registry: not applicable.
2. Unique identifying number of the study: not applicable.
3. Hyperlink to your specific registration (must be publicly accessible and will be checked): not applicable.

## Guarantor

Saurav Agrawal is the guarantor.

## Data availability statement

Not applicable.

## Provence and peer review

Not commissioned, externally peer-reviewed.
